# Rare occurrence of lepra type 1 reaction in pure neuritic leprosy: A case report

**DOI:** 10.1002/ccr3.4324

**Published:** 2021-06-24

**Authors:** Raksha Pathak, Sudha Agrawal, Punam Paudyal

**Affiliations:** ^1^ Department of Dermatology and Venereology B.P Koirala Institute of Health Sciences Dharan Nepal; ^2^ Department of Pathology B.P Koirala Institute of Health Sciences Dharan Nepal

**Keywords:** pure neuritic leprosy, type 1 reaction

## Abstract

Type 1 reaction in pure neuritic leprosy usually occurs in the form of neuritis. The development of new skin lesion during reaction is rare. Clinicians should be aware about occurrence of type 1 reaction in pure neuritic leprosy.

## Graphical abstract

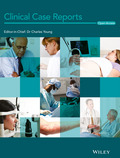

## 1 INTRODUCTION

Pure neuritic leprosy as a distinct type of leprosy is recognized only under Indian Association of Leprologist's classification. It includes leprosy cases affecting peripheral nerves without any skin lesions.^1^ Type 1 reactions (reversal reactions) in leprosy patients present as increased inflammation of preexisting lesions, appearance of new erythematous lesions, facial and pedal edema, and even neuritis.^2^ Occurrence of type 1 reaction is not common in pure neuritic leprosy. Whenever it occurs, it usually manifests as increased intensity of neuritis and nerve function impairment. However, new skin lesion as manifestation of reactional state is unusual.^3^ We present a case of an adult female diagnosed with pure neuritic leprosy, who later developed type 1 reaction in the form of neuritis, pedal edema, and appearance of erythematous and edematous new skin lesion over the left cheek. Type 1 reaction was confirmed by histopathology, and the patient was successfully managed with WHO MB‐MDT and oral corticosteroids.

## 2 CASE REPORT

**FIGURE 1:**
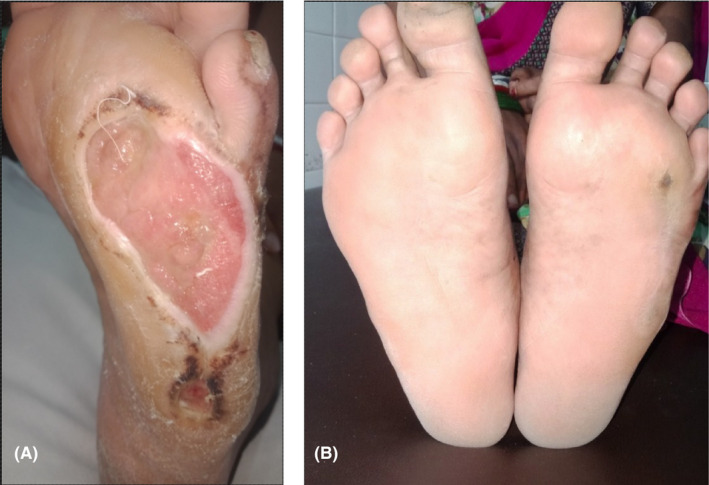
A, Single well‐defined 5 × 3 cm^2^ ulcer over planter aspect of left foot with sloping edge, hyperkeratotic margin, and floor with healthy granulation tissue. B, Healed ulcer after 4 wk

A 36‐year‐old lady presented with ulcer over anterolateral aspect of left foot (Figure 1A) for a duration of 8 months. She was diagnosed as leprosy from other center and was started on WHO MB‐MDT blister pack. She was under fifth blister pack at the time of presentation to our center. She had history of decreased sensation over bilateral hands and feet but there was no history of any cutaneous lesions of leprosy.

Clinical examination showed thickened and non‐tender peripheral nerves (ulnar, radio cutaneous, and lateral popliteal) with complete loss of sensation to temperature (hot/cold), touch, and pain over bilateral hands and feet as per WHO testing sites. There was hypothenar atrophy on right hand and mobile clawing of little finger on left hand. No cutaneous lesions suggestive of leprosy were noted.

Slit skin smear was negative. Nerve conduction study revealed asymmetrical sensory‐motor axonal polyneuropathy involving peripheral nerves of upper limbs and symmetrical sensory involvement on nerves of lower limbs. The diagnosis of pure neuritic Hansen's disease with grade II disability of bilateral hands and left foot and grade I disability of right foot was made, and the patient was continued with WHO MB‐MDT. For trophic ulcer, conventional dressing was done daily with ointment mupirocin followed by application of paraffin mesh (Jelonettm Smith & Nephew) and non‐adherent dressing. The ulcer was healed completely in 4 weeks (Figure 1B).

**FIGURE 2:**
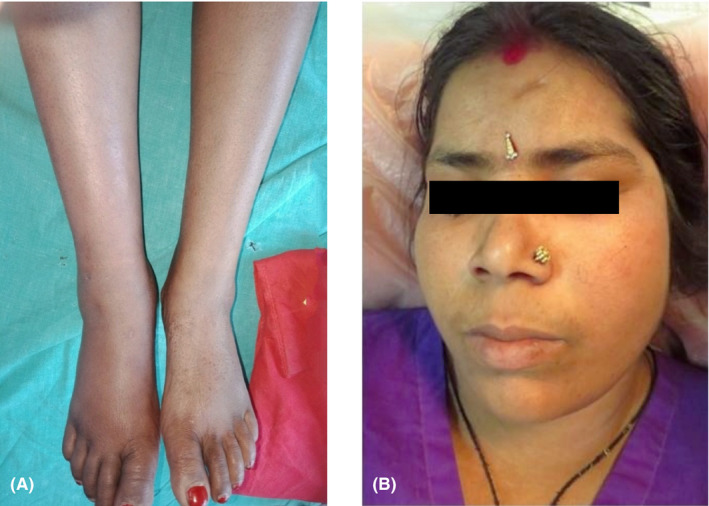
A, Edema over bilateral legs and feet. B, Erythematous edematous area on left cheek

Three months later, the patient developed acute onset swelling of bilateral legs associated with tingling sensation disturbing daily activities. There was presence of pitting edema over bilateral feet extending till distal one‐third of legs (Figure 2A). Similarly, erythematous edematous area was noticed over left cheek (Figure 2B).

**FIGURE 3:**
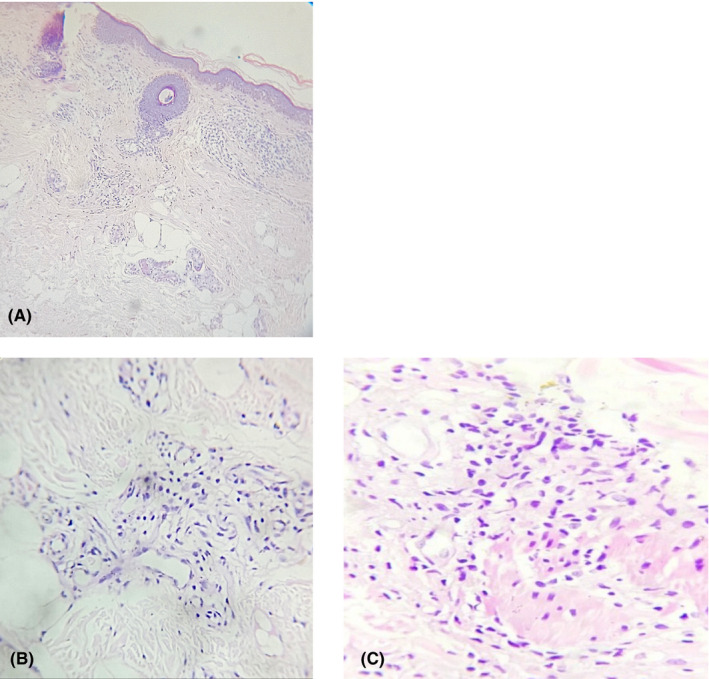
A, Histopathology showing perivascular, periadnexal, and interstitial infiltrates in reticular dermis along with dermal edema suggesting lepra type 1 reaction (H and E stain 10×). B, Perivascular lymphohistiocytic infiltrates with few epithelioid cells and edematous stroma (H and E stain 40×). C, Infiltrates encroaching arrector pili muscles and destructing nerve bundles (H and E stain 40×)

The clinical diagnosis of lepra type 1 reaction was confirmed by examination of biopsy specimen taken from two sites, face and leg, which showed edematous dermis with perivascular, periadnexal, and interstitial lymphohistiocytic infiltrate along with few epithelioid histiocytes. The infiltrates were encroaching the arrector pili muscles and destructing nerve bundles. Stain for acid‐fast bacilli (lepra) was negative (Figure 3A‐C).

**FIGURE 4:**
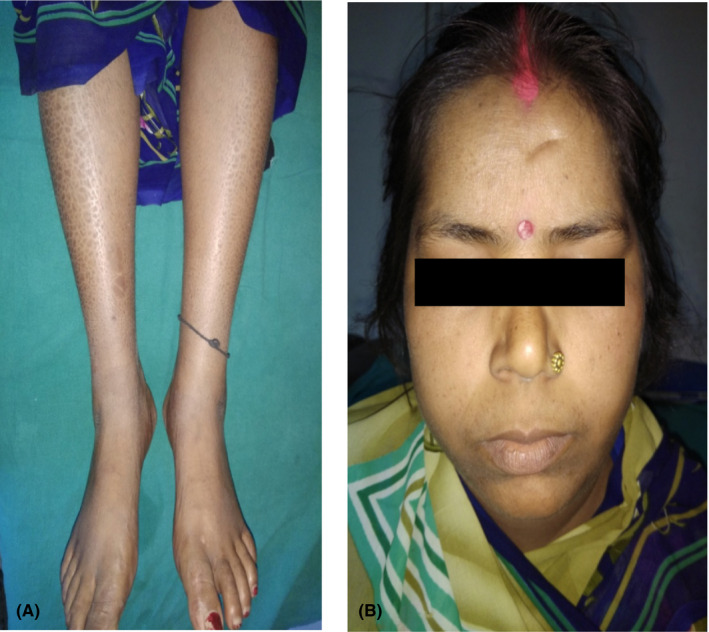
A, After treatment with oral corticosteroids, edema of legs and feet subsided with ichthyosis over bilateral legs. B, Erythema and edema on left cheek resolved

Erythema and edema over left cheek and edema on bilateral legs improved considerably after 2 weeks of starting oral prednisolone 40 mg following which dose of corticosteroid was tapered slowly. The pedal edema subsided (Figure 4A) and the erythematous plaque over the left cheek resolved (Figure 4B) in 8 weeks, and after 30 weeks of treatment with tapering dose of oral corticosteroid, steroid was stopped. The patient completed 24 blister pack of WHO MB‐MDT.

## 3 DISCUSSION

Pure neuritic leprosy is an uncommon presentation of leprosy. However, in Indian subcontinent, 4.6%‐17.7% of leprosy patients present with nerve involvement without skin lesions.^4^ Sensory, motor, and/or autonomic deficit occurs along the distribution of the affected nerve.^5^ Ulnar nerve and lateral popliteal nerve are the most common nerves involved in upper limbs and lower limbs, respectively, though, any nerve can be involved.^3^ They are frequently misdiagnosed because of absence of skin lesions and negative slit skin smear and can present with grade II disability as initial presentation.^6^ Nerve biopsy is the gold standard for diagnosis but it is an invasive procedure with possible complications like nerve damage. The non‐invasive high‐quality electrophysiological studies are helpful in the diagnosis of leprosy.^7^

On follow‐up, 15%‐35% of pure neuritic leprosy patients develop visible skin lesions which may indicate prolonged neuritic phase preceding the appearance of cutaneous lesions and most of these lesions are confirmed as borderline tuberculoid type histopathologically.^8^, ^9^, ^10^, ^11^ Sudden appearance of new lesions can occur due to fluctuations of cell‐mediated immunity in untreated or treated cases of leprosy. Some authors considered that the new skin lesions developed were possibly manifestation of reversal reaction.^9^, ^10^, ^11^, ^12^ Absence of visible skin lesions in patients is probably related to the deep location of granuloma in the dermis^13^ which could be manifested during reactions. This is yet to be proven. Due to absence of preexisting lesions to manifest inflammatory process, the diagnosis of type 1 reaction in pure neuritic leprosy is often missed, under‐recorded, and under‐reported.^3^

In our patient, the diagnosis of pure neuritic leprosy was made by clinical examination performed by an experienced clinician combined with nerve conduction studies and the type 1 lepra reaction by increased tingling sensation with the development of edematous and erythematous new lesion over left cheek and pedal edema. This was correlated histopathologically with biopsy from these sites and resolution of pedal edema and lesion over the cheek after administration of oral corticosteroids. The clinicians should be aware about unusual presentation of type 1 reaction in pure neuritic leprosy so that unnecessary workups are avoided and disabilities are prevented.

## CONFLICT OF INTEREST

None declared.

## AUTHOR CONTRIBUTION

RP: Wrote the manuscript and reviewed literature. SA: Involved in concept, manuscript editing, guidance, and approval of the final version of the manuscript. PP: Involved in histopathological reporting.

## ETHICAL APPROVAL

Written consent was taken from the patient for the publication of the case report and the images.

## Data Availability

We agree to make the manuscript available to general people and are also ready to provide other necessary data regarding the manuscript in case required.
